# On the Maternal Management of Children in Health and Disease

**Published:** 1849-09

**Authors:** 


ART. VI.—*On the Maternal Management of Children in Health and Disease.*—By THOMAS BULL, M. D., Member of the Royal College of Physicians. From the third London Edition; Philadelphia; Lindsay & Blakiston; 1849; pp. 506., 12 mo. (From the Publishers. For sale by S. C. Griggs & Co.)

This is an excellent little book—the best of its kind we have seen. As the title indicates, it is not designed for the practitioner, but for the mother of the family. There are many matters connected with the rearing of children, with which every mother should be familiar; but of which unfortunately, the majority of women are totally ignorant. The health of children—and the remark applies with peculiar force to girls—is often ruined [illegible] and indiscretion of the mother. This evil, the little volume before us is designed to obviate, by giving judicious general directions with regard to the management of children in health and disease.—Heretofore the education of women has been wofully defective. They have been taught music, painting, embroidery, &c., the mint, anise and cummin of a good education; but of the weightier matters of the law—the laws of life—the means of securing sound constitutions—a knowledge of the duties and responsibilities they are to assume in after life, they are taught little or nothing.

For this defect, this little volume is intended to compensate. The work treats fully of the general management of children in health and disease.

The directions are judicious and valuable; and we can cheerfully recommend the book to the favorable attention of the profession and the community at large. It contains some *Anglicisms* which are not applicable to this meridian; but these do not detract to any considerable extent from the value of the volume. We hope our subscribers will buy it for themselves, and recommend their patrons to do the same. It will be money well invested. M.

